# Retraction: MicroRNA-433 inhibits oral squamous cell carcinoma cells by targeting FAK

**DOI:** 10.18632/oncotarget.28270

**Published:** 2022-09-14

**Authors:** Yong-Jian Wang, Zi-Feng Zhang, Shao-Hua Fan, Juan Zhuang, Qun Shan, Xin-Rui Han, Xin Wen, Meng-Qiu Li, Bin Hu, Chun-Hui Sun, Bin Qiao, Qian Tao, Dong-Mei Wu, Jun Lu, Yuan-Lin Zheng

**Affiliations:** ^1^Key Laboratory for Biotechnology on Medicinal Plants of Jiangsu Province, School of Life Science, Jiangsu Normal University, Xuzhou 221116, P.R. China; ^2^School of Environment Science and Spatial Informatics, China University of Mining and Technology, Xuzhou 221008, P.R. China; ^3^Jiangsu Key Laboratory for Eco-Agricultural Biotechnology around Hongze Lake, School of Life Sciences, Huaiyin Normal University, Huaian 223300, P.R. China; ^4^Department of Oral and Maxillofacial Surgery, Guanghua School and Hospital of Stomatology, Guangdong Provincial Key Laboratory of Oral Diseases, Sun Yat-Sen University, Guangzhou 510055, P.R. China; ^*^These authors have contributed equally to this work

**This article has been retracted:** In July of 2020, the Academic Committee of Jiangsu Normal University launched an investigation into this paper and requested that the authors provide the original experimental records and data for verification. To date, however, the authors have failed to provide the requested documents. The committee has concluded that this paper displays signs of academic fraud. In light of this, the paper is retracted. The University and Oncotarget thank the Pubpeer community for raising the concerns.

Original article: Oncotarget. 2017; 8:100227–100241. 100227-100241. https://doi.org/10.18632/oncotarget.22151

